# Hypoglycemic, hypolipidemic and antioxidant effects of pioglitazone, insulin and synbiotic in diabetic rats

**DOI:** 10.14202/vetworld.2016.118-122

**Published:** 2016-02-03

**Authors:** K. Kavitha, A. Gopala Reddy, K. Kondal Reddy, C. S. V. Satish Kumar, G. Boobalan, K. Jayakanth

**Affiliations:** 1 Department of Veterinary Pharmacology and Toxicology, College of Veterinary Science, Hyderabad - 500 030, Telangana, India; 2 Department of Livestock Products Technology, College of Veterinary Science, Hyderabad - 500 030, Telangana, India

**Keywords:** diabetes, insulin, oxidative stress, pioglitazone, synbiotic

## Abstract

**Aim:**

The objective of the study was to assess the effect of combination treatment of insulin, pioglitazone and synbiotic on streptozotocin (STZ)-induced diabetic rats.

**Materials and Methods:**

Diabetes mellitus was induced chemically by intraperitoneal administration of STZ (40 mg/kg b.wt) to male Sprague-Dawley rats. The rats were divided randomly into six groups of six rats in each. Group 1 was maintained as a normal control. Group 2 was maintained as diabetic control; Group 3 was treated with insulin; Group 4 with insulin + synbiotic; Group 5 with insulin + pioglitazone; and Group 6 with insulin + synbiotic + pioglitazone. All the animals were treated for 60 days.

**Results:**

Body weights, and concentration of reduced glutathione (GSH), and high-density lipoproteins cholesterol were significantly (p<0.05) reduced, whereas the concentration of blood glucose, total cholesterol, triglycerides, protein carbonyls and thiobarbituric acid reacting substances, and the activity of GSH peroxidase were significantly (p<0.05) elevated in Group 2 at the end of 8^th^ week as compared to Group 1. The treatment Groups 3, 4, 5 and 6 revealed improvement in all the parameters, and the highest improvement was observed in combination Group 6.

**Conclusion:**

From this study, it is concluded that combination of insulin, pioglitazone and synbiotic is useful in treating diabetes.

## Introduction

Diabetes mellitus (DM) is a metabolic disease characterized by insulin resistance, dyslipidemia, and hyperglycemia. Insulin resistance and beta-cell dysfunction are the pathophysiological hallmarks of Type 2 DM [1]. Increase in oxidative stress and changes in antioxidant capacity, induced by high glucose, play a central role in complications of diabetes [2]. Pioglitazone is a potent insulin sensitizer, binds to the peroxisome proliferator-activated receptor gamma (PPARγ) and enhances the sensitivity of liver, muscle and adipose tissue to insulin by promoting the uptake of glucose. It has been reported that pioglitazone improves plasma lipid profile and blood pressure, which are very beneficial for the diabetic patients associated with cardiovascular diseases [3].

Prebiotics is food ingredients, such as dietary fibers consisting of non-starch polysaccharides and lignin that are non-digestible and promote the growth of specific bacteria within the intestinal tract that confer health benefits in the host. They are used in medicine as a means of providing short chain fatty acids (SCFA), maintaining bowel function, building colonization resistance against pathogens, and treating antibiotic-associated diarrhea as well as inflammatory bowel disease [4]. Prebiotic increases the endogenous intestinotrophic proglucagon-derived peptide (GLP-2) production and consequently improves gut barrier functions during obesity and diabetes [5]. Probiotics are live microbial organisms confers health benefits to the host by interacting with intestinal mucosal barrier and potentially contributes to the homeostasis of mucosal immunity. *Lactobacillus* and *Bifidobacteria* species are the most common genera used in fermentation of foodstuffs as probiotics. Synbiotic is a combination of probiotic and prebiotic administered together. The administration of synbiotic may be beneficial as adjunct therapy in the treatment of diabetes [6].

The aim of the present study was to assess the combined effect of insulin, pioglitazone and synbiotic on blood glucose concentration, lipid profile, and antioxidant profile in diabetic rats.

## Materials and Methods

### Ethical approval

An experimental protocol was conducted on rats with the approval from Institutional Animal Ethics Committee (No. 2/I/2013).

### Drugs and chemicals

Insulin (Insuman Basal-Aventis) was administered as a subcutaneous injection. Pioglitazone (Dr. Reddy's, India) was administered as a suspension in freshly prepared in 0.5% w/v carboxymethyl cellulose. Synbiotic, a combination of probiotic (*Bifidobacterium bifidum* 231) was procured from Department of Livestock Products Technology, College of Veterinary Science, Hyderabad, India, and prebiotic (fructo oligosaccharide [FOS]) from Xena Bioherbals, India were administered in normal saline.

### Animals

A total of 36 male Sprague-Dawley rats were procured from the National Centre for Laboratory Animal Sciences, National Institute of Nutrition, Hyderabad, India. The rats were equally divided into six groups and acclimatized for a period of 2-week. Streptozotocin (STZ) at 40 mg/kg body weight was administered intraperitoneally to induce diabetes in five groups. A control group was administered with the normal saline. Blood samples were collected after 72 h and serum was separated to estimate serum glucose level. Rats with blood glucose value of >200 mg/dl were included in the study.

### Experimental design

All the rats in groups were maintained as per the following treatment schedule for 8 weeks. Group 1: Non-diabetic control; Group 2: STZ (40 mg/kg i/p single dose)-induced diabetic control; Group 3: Insulin (4 U/kg once daily for 8 weeks) treatment in diabetic rats; Group 4: Insulin + synbiotic treatment (combination of *B. bifidum* 231 at 1.4 × 10^11^ CFU/rat/day intragastrically twice daily and FOS at 0.8 g/rat/day once daily for 8 weeks) in diabetic rats; Group 5: Insulin + pioglitazone (10 mg/kg once daily for 8 weeks) treatment in diabetic rats; and Group 6: Insulin + pioglitazone + synbiotic treatment in diabetic rats. The doses were selected based on in-house experimental data and literature search.

### Blood and tissue collection

Blood was collected once in 2 weeks into serum vacutainers through retro-orbital plexus. The serum was separated and stored at −20°C and tested for estimation of glucose (fortnightly), and total cholesterol (TC), triglycerides (TGs), and high-density lipoprotein cholesterol (HDL-C) concentration by using kits (Sigma Diagnostics Pvt. Ltd., India) at the end of the 4^th^ and 8^th^ week. Body weights of all groups were recorded at fortnight intervals. At the end of the study, six rats from each group were sacrificed and kidneys were collected, snap frozen and stored at −20°C for further estimation of reduced glutathione (GSH) [7], GSH peroxidase (GPx) [8], thiobarbituric acid reacting substances (TBARS) [9], and protein ­carbonyls [10] in kidney homogenates.

### Statistical analysis

The data were analyzed by one-way ANOVA using statistical package for social sciences (SPSS; version 16.0). Differences between means were tested using Duncan's multiple comparison tests and a significance was set at p<0.05.

## Results

The mean body weight in diabetic control Group 2 was significantly (p<0.05) lower as compared to all the other groups on day 30 and 60. The body weights in the Groups 3-6 were significantly (p<0.05) higher as compared to Group 2 at the end of day 30 and 60. The Groups 5 and 6 showed a significant (p<0.05) increase in the body weights among all the treated groups at the end of the 60^th^ day (Table-1). The serum glucose concentration (mg/dl) in the Group 1 was significantly (p<0.05) lower, whereas Group 2 showed significantly (p<0.05) higher concentration as compared to other groups throughout the experiment. All the treated Groups (3-6) showed significant (p<0.05) decrease in glucose concentration on day 30 and 60 as compared to Group 2; the decrease was the highest in Groups 5 and 6 (Table-1).

**Table-1:** Body weight (g) and serum glucose concentration (mg/dl) in different groups of rats.

Groups (G)	Body weight (g)		Serum glucose (mg/dl)	
	
	30^th^ day	60^th^ day	30^th^ day	60^th^ day
G 1	347.17±8.73^d^	406.83±6.31^e^	80.67±2.21^a^	83.17±1.83^a^
G 2	235.17±6.65^a^	203.33±4.14^a^	299.00±1.06^d^	312.17±5.75^e^
G 3	302.83±9.77^b^	330.67±10.36^b^	241.50±3.93^c^	211.17±3.19^d^
G 4	307.17±7.21^bc^	332.50±7.83^b^	240.50±2.78^c^	200.70±3.97^cd^
G 5	313.33±3.02^bc^	351.17±3.89^c^	238.17±3.51^bc^	191.17±6.30^c^
G 6	326.33±5.13^c^	381.00±3.15^d^	229.33±5.87^b^	165.50±3.13^b^

Values are mean±SE (n=6); Duncan’s multiple comparison test, means with different alphabets as superscripts differ significantly (p<0.05). SE=Standard error

The concentration of TC and TG (mg/dl) in Group 2 was significantly (p<0.05) higher on day 30 and 60, as compared to Group 1. The values of the above variables in the treatment Groups (3-6), were significantly (p<0.05) reduced at the end of the 60^th^ day as compared to Group 2. The HDL-C concentration (mg/dl) was significantly (p<0.05) lower in Group 2 as compared to Group 1 on day 30 and 60, while the treatment Groups 3, 4, 5 and 6 showed significant (p<0.05) increase in HDL-C (Table-2).

**Table-2:** Serum lipid profile in different groups of rats.

Groups (G)	TC (mg/dl)		TGs (mg/dl)		HDL-C (mg/dl)	
		
	30^th^ day	60^th^ day	30^th^ day	60^th^ day	30^th^ day	60^th^ day
G 1	135.29±1.00^a^	138.29±1.00^a^	77.10±0.27^a^	76.03±0.66^a^	55.33±0.22^f^	57.69±0.49^f^
G 2	280.95±1.35^e^	309.53±1.31^f^	113.75±0.30^e^	123.56±0.30^e^	41.23±0.86^a^	42.01±0.94^a^
G 3	159.43±2.14^d^	151.81±0.70^e^	94.34±0.57^d^	89.70±0.27^d^	43.36±0.44^b^	45.11±0.49^b^
G 4	153.80±1.29^c^	147.17±0.47^d^	90.03±0.28^c^	86.31±0.60^c^	45.36±0.32^c^	47.16±0.43^c^
G 5	154.34±1.39^c^	143.29±1.03^c^	82.38±0.64^b^	81.14±0.77^b^	47.02±0.47^d^	49.89±0.45^d^
G 6	149.44±0.65^b^	139.17±1.35^b^	83.34±0.94^b^	77.01±0.46^a^	48.92±0.24^e^	54.34±0.76^e^

Values are mean±SE (n=6); Duncan’s multiple comparison test, means with different alphabets as superscripts differ significantly (p<0.05). SE=Standard error, TC=Total cholesterol, TG=Triglycerides, HDL-C=High density lipoprotein cholesterol

The parameters of oxidant-antioxidant status in kidney revealed significant (p<0.05) increase in the activity of GPx, and the concentration of TBARS and protein carbonyls, while the concentration of GSH was significantly (p<0.05) lowered in Group 2. The values of these parameters revealed an improvement in the treatment Groups 3, 4, 5 and 6 at the end of the 60^th^ day (Table-3).

**Table-3:** Anti-oxidant profile in kidney homogenates in different groups of rats.

Groups (G)	GPx (U/mg protein)	TBARS (nmol of MDA/mg protein)	Protein carbonyls (nmol carbonyls/mg protein)	GSH (nmol/mg protein)

	60^th^ day	60^th^ day	60^th^ day	60^th^ day
G 1	0.61±0.02^a^	3.53±0.06^a^	2.42±0.07^a^	30.51±0.24^f^
G 2	1.26±0.03^f^	8.59±0.09^f^	6.34±0.11^f^	15.05±0.31^a^
G 3	1.06±0.02^e^	6.16±0.05^e^	3.58±0.06^e^	23.30±0.39^b^
G 4	0.92±0.02^d^	4.91±0.05^d^	3.49±0.03^cd^	24.80±0.45^c^
G 5	0.85±0.02^c^	4.61±0.05^c^	3.31±0.06^bc^	26.52±0.38^d^
G 6	0.74±0.02^b^	4.37±0.06^b^	3.23±0.03^b^	27.64±0.26^e^

Values are mean±SE (n=6); Duncan’s multiple comparison test, means with different alphabets as superscripts differ significantly (p<0.05). SE=Standard error, GPx=Glutathione peroxidase, TBARS=Thiobarbituric acid reacting substances, GSH=Glutathione

## Discussion

The cytotoxic action of STZ is mediated by the formation of free radicals such as superoxide and hydroxyl radicals that can cause rapid destruction of β-cells of the pancreas, resulting in partial or complete loss of insulin production, thereby resulting in the development of hyperglycemia and its complications, if untreated [11]. Body weights were significantly decreased in diabetic control may be due to catabolic processes involved in DM.

Thiazolidinediones like pioglitazone binds to PPARγ receptor promotes glucose uptake by increasing expression of insulin receptor substrate-2 and glucose transporter 4 protein in insulin sensitive tissues *viz*. adipose tissue, muscle and liver, results decrease in postprandial and fasting plasma glucose levels [12]. Pioglitazone increase body weight through *in vivo* up-regulation of genes including phosphoenolpyruvate carboxykinase, glycerol-3-phosphate dehydrogenase, and acetyl-CoA synthetase facilitating adipocyte lipid storage pathways [13].

Treatment with synbiotic reduce elevated blood glucose levels may be by stimulation of peripheral tissues glucose uptake, alteration of insulin metabolism and inhibition of glucose reabsorption by the kidneys, resulting in the elimination of glucose in the urine as reported by Roselino *et al*. [14]. Treatment with a combination of insulin, pioglitazone and synbiotic improved the body weights, and significantly lowered serum glucose concentration compared to other treated groups.

Rise in plasma TG and TC, and fall in HDL-C in diabetic animals is due to increased lipolysis in adipose tissue, and decreased the activity of insulin-dependent lipoprotein lipase leading to elevated blood levels of fatty acids, which are available for the synthesis of TG. Pioglitazone enhances lipoprotein lipase activity and insulin sensitivity by increasing the expression and differentiation of adiponectin resulting in reduced TG levels and increased serum HDL-C levels [15, 16]. This may provide another explanation for the improvement in insulin sensitivity despite weight gain by pioglitazone.

Synbiotics improves lipid profile in diabetic animals by producing SCFA, carbon disulfide and methyl acetate and can increase the lipolytic activity [17]. Based on previous *in vitro* and *in vivo* trials, probiotics accounted for serum cholesterol reduction by improving lipid profiles. Probiotics controls cholesterol metabolism by several ways: (1) They removes cholesterol by assimilation in the small intestine, (2) probiotics reduces absorption of cholesterol in the intestine which in turn reduces the serum cholesterol, and (3) probiotics incorporates the cholesterol into cell membranes. The current study results are in consistent with Liong *et al*. [18], stated that intake of synbiotic for 8 weeks resulted in a decreased serum TG, TC levels and increased HDL-C concentrations in hypercholesterolemic pigs. In Group 6, the administration of insulin, pioglitazone and synbiotic revealed a significant alteration in lipid profile to normal.

In the diabetic control group, the GPx activity, and the TBARS and protein carbonyls levels were significantly increased with a significant decrease in GSH level. These findings indicate ongoing free radical-induced oxidative damage in the living system by autoxidative glycosylation, hence depriving the antioxidant defenses. The antioxidant activity of pioglitazone is reported to be mediated by blocking the vicious cycle of reactive oxygen species production, enhancing the insulin sensitivity and arrest the proinflammatory signaling transduction [19, 20]. The probiotic *B. bifidum* present in synbiotic has defense mechanisms, exerts by modulating the mucosal immune system by blocking the proinflammatory cytokines, inhibit the attachment of pathogenic bacteria by producing antibacterial compounds and enhance the protective function of epithelial cells, ability to capture and make free radicals harmless and maintains homeostasis of digestive tract thereby reducing the oxidant levels and acts as antioxidants [21]. Treatment with insulin, pioglitazone and synbiotic in Group 6 resulted in the revival of antioxidant defenses toward normal, which confirms synergistic antioxidant potential of pioglitazone and synbiotic.

## Conclusion

In summary, the present study demonstrated that combination of insulin, pioglitazone and synbiotic could significantly improve blood glucose concentration, improves lipid profile, and decrease oxidative stress in diabetic rats as compared to other treatments tested in this experiment, which was evident from the significant reversal of biochemical changes.

## Authors’ Contributions

This study is the major component of the work toward the PhD thesis of KK, under the guidance of the second author AGR. KK conducted the experiment and organized the manuscript, and AGR and KKR thoroughly revised the same. CSVSK, GB, JK assisted in housing and maintenance of rats, blood collection, organ collection, data analysis and interpretation. All authors read and approved the final manuscript.
